# Hole‐in‐the‐head disease in discus fish, *Symphysodon* (Heckel, 1840): Is it a consequence of a dietary Ca/P imbalance?

**DOI:** 10.1111/jfd.13023

**Published:** 2019-05-26

**Authors:** Alessandra Amesberger‐Freitag, Alexander Tichy, Mansour El‐Matbouli, Eva Lewisch

**Affiliations:** ^1^ Clinical Division of Fish Medicine University of Veterinary Medicine Vienna Austria; ^2^ Bioinformatics and Biostatistics Platform University of Veterinary Medicine Vienna Austria

**Keywords:** beef heart, Ca/P ratio, calcium, hole‐in‐the‐head disease, phosphorus, *Symphysodon*

## Abstract

Hole‐in‐the‐head (HITH) disease‐affected fish develop characteristic lesions in the skin above sensory pores of the head and the trunk. This study investigated whether an unfavourable Ca/P ratio in the diet could provoke lesions consistent with HITH disease in discus fish *Symphysodon* (Heckel, 1840) as a comparable condition to secondary hyperparathyroidism of tetrapod species. Two groups of five fish were fed a plain beef heart diet (Ca/P of 0.03), whereas two other groups were kept on commercial discus feed (Ca/P of 2.73). Each feeding group was submitted to two different water hardness regimes (35.66–71.39 mg/L CaCO_3_ and 124.94–196.33 mg/L CaCO_3_, respectively). All fish were observed for the development of the characteristic lesions for 16 weeks. At the end of the study, histological, bacteriological and parasitological examinations were conducted and plasma Ca, P and Mg values were determined. Diplomonad flagellates were detected in two fish. Isolated bacteria of all groups mostly belonged to Aeromonadales and Pseudomonadales. No significant difference of plasma mineral values between the groups was observed. Compared to the results of other authors, Ca stayed mainly in the range and P exceeded the reference values. Histological examinations did not indicate HITH disease, and no fish developed signs of the disease during the study.

Clinical trial registration number GZ 68.205/0135‐WF/V/36/2014.

## 1 INTRODUCTION

A characteristic occurrence of skin depigmentation and erosion involving the sensory pores of the head and the lateral line has been reported in several families of marine and freshwater fish (Corrales, Ullal, & Noga, 2009; Gratzek, 1988; Hemdal, 2006; Noga, 2010). Commonly referred to as head and lateral line erosion (HLLE) by hobbyists, other names like hole‐in‐the‐head (HITH) disease or lateral line depigmentation (LLD) are used depending on the main signs. The condition not only leads to a disfiguration of the fish, but also can develop to a severe and finally lethal disease. Among cichlids, discus fish (*Symphysodon* sp.) seem to be especially susceptible (Noga, 2010). Both wild‐caught and commercially farmed discus fish are highly valued by professional breeders and hobbyists and are placed in the upper price segment of the ornamental fish industry, which makes affected fish especially undesirable.

Despite a considerable variety of infectious, nutritional and environmental conditions has been discussed to cause the signs, the underlying aetiology is still unclear. Information relies mostly on magazines for aquarium hobbyists, and scientific experiments on the subject are scarce. Most of the authors mentioned below agree that unfavourable and stressful environmental conditions may play a role in the development of the disease. Among the proposed specific causes, Paull and Matthews (2001) described *Spironucleus vortens* as the possible causative agent. Other authors attributed the disease to bacterial or viral infections (Bullock & Herman, 1985; Hemdal, 1989; Varner & Lewis, 1991). Also, different water compositions (Untergasser, 1991; Noga, 2000; Pro, 2005; Roberts & Palmeiro, 2008; Katharios et al., 2011), the use of activated carbon (Stamper, Kittell, Patel, & C. A. L., 2011), stress (Bartelme, 2003a) and genetic predispositions (Bartelme, 2003b) have been suggested as a cause of the disease. Nutritional imbalances including vitamin C deficiencies (Collins, 1995) and mineral imbalances (Noga, 2010) have also been suggested causing HITH disease.

A feeding regime exemplary for such mineral imbalance is the use of raw beef heart as a source of protein, which was suggested by Chong, Hashim, Chow‐Yang, and Ali (2002) for commercial discus farming. The authors found good digestibility values for dry matter and protein of the raw beef heart, resulting in accelerated growth and intense coloration of the fish. Thus, feeding beef heart to discus has become very popular among breeders and hobbyists, often with no or insufficient supplementation (Untergasser, 1991). Nevertheless, Ca contents of beef heart are far below suggested for the diet of the comparable species *Cichlasoma urophthalmus* (0.6 g/kg vs. 1.8 g/kg), while P is exceeding recommendations (20.6 g/kg vs. 1.5 g/kg), leading to a Ca/P of 0.03, while ratios of 1.3 are recommended (Chavez‐Sanchez, Martinez‐Palacios, Martinez‐Perez, & Ross, 2000). Such low‐Ca and high‐P diets are known to cause alimentary secondary hyperparathyroidism in non‐aquatic species like horses (Lacitignola et al., 2018), rabbits (Bas et al., 2005), dogs (Kawaguchi, Braga, Takahashi, Ochiai, & Itakura, 1993), reptiles (Mans & Braun, 2014), cats (Tomsa et al., 1999) and birds (Wallach & Flieg, 1969). This condition is caused by calcium deficiency or an imbalance of Ca/P in the diet and leads to hypocalcaemia, which causes increased release of parathyroid hormone, resulting in chronic bone resorption.

In teleost fish, the role of parathyroid hormone‐related protein (PTHrP) for Ca and P metabolism was demonstrated for some fish species (Abbink, 2004; Abbink & Flik, 2007; Guerreiro, Renfro, Power, & Canario, 2007). Abbink and Flik (2007) suggest a role of PTHrP for Ca mobilization from bones and scales while Witten and Huysseune (2009) attribute bone resorption rather to a lack of P than to Ca deficiency. These authors also state that the teleost endoskeleton is not used as a source of minerals when in demand. On the other hand, Takagi and Yamada (1993) showed that in tilapia (*Oreochromis niloticus*) Ca depletion led to bone resorption and decreased bone Ca and P contents. While the endocrine mechanisms regulating the Ca/P balance differ substantially from terrestrial animals, decreasing Ca/P in the diet of carp (*Cyprinus carpio*) was demonstrated to decrease the Ca absorption rate significantly (Nakamura & Yamada, 1980). Until now, evidence of bone resorption or involvement of an impaired Ca and P metabolism in HITH disease has not been described.

This study was conducted to determine whether feeding a plain beef heart diet would cause HITH disease in discus fish as a result of an altered osseous metabolism comparable to secondary hyperparathyroidism of tetrapods. Furthermore, a possible correlation between the selected dietary regimes, water parameters and plasma values of the concerned minerals should be evaluated.

## 2 MATERIALS AND METHODS

This study was approved by the Animal Experimentation Ethics Committee of the University of Veterinary Medicine in Vienna and from the Ministry of Science of Austria according to § 26 of the Austrian law for animal experiments under the number GZ68.205/0135‐WF/V/36/2014 conforming to Directive 2010/63/EU.

Twenty‐four discus fish (captive bred, mixed gender, mean length 11.7 ± 4.5 cm, mean weight 57.45 ± 52.8 g) from a commercial breeding facility were assigned at random to four groups (A–D) of six animals each. One fish of each group was killed and sampled upon arrival as described below to serve as a reference (Ref), while the other fish were acclimated for one week. During acclimatization, the groups were maintained in 250‐L recirculation tanks in filtered and UV‐treated well water with pH 7.0 and 124.9–196.3 mg  L CaCO_3. _Each tank was filtered individually, and temperature was set to 30°C. During that time, all fish were fed a commercial discus diet (JBL Grana Discus®, JBL). After one week of acclimatization, two groups were designated as experimental groups (A and C) and two as controls (B and D). Water CaCO_3_ content in one experimental (A) and one control group (B) was changed to 35.7–71.4 mg L CaCO_3_. Diet was switched to beef heart in the experimental groups (A and C), whereas the commercial feed was retained in the control groups (B and D) (Table 1). All groups were fed twice a day at 2% of their body weight. The raw beef heart diet was prepared by removing all tendons and fat from the heart, grinding the meat, and stored at −20°C. Diet contents of Ca, P and Mg were analysed by the Austrian Agency for Health and Food Safety, Ltd. (AGES).

**Table 1:** Allocation of fish to the different treatment groups

Group	Water CaCO_3_ mg/L	Diet	Fish						
			f/m	Start		End		LGR %	WGR %
				BL (cm) (mean, range)	BW (g) (mean, range)	BL (cm) (mean, range)	BW (g) (mean, range)		
Ref *n* = 4	124.9–196.3	Commercial	0/4	13.1 ± 2	79.7 ± 49	–	–	–	–
A *n* = 5	35.7–71.4	Beef heart	2/3	12.8 ± 3	74.28 ± 25.3	13.4 ± 2.5	81.01 ± 27.6	4.7	9.1
B *n* = 5	35.7–71.4	Commercial	0/5	11.2 ± 2	45.6 ± 25.4	11.9 ± 2	49.18 ± 23.4	6.3	7.9
C *n* = 5	124.9–196.3	Beef heart	3/2	11.2 ± 2	49.93 ± 23.0	11.8 ± 2	55.82 ± 26.5	5.3	11.8
D *n* = 5	124.9–196.3	Commercial	2/3	10.6 ± 2	40.48 ± 30.2	11.2 ± 2.5	43.56 ± 35.7	5.7	7.6

Note

Body lengths (BL) and weights (BW) are given for the start and the end of the study, except for reference fish (Ref), which were killed upon arrival.

Abbreviations: f, female; LGR, length gain rate; m, male; *n*, number of fish; WGR, weight gain rate.

Carbonate hardness, as well as temperature and pH, was controlled daily. Temperature and pH were determined using a multiparameter portable meter (WTW multi 3420, Xylem Analytics). Water CaCO_3_ content was calculated daily from the results of complexometric hardness titration with hydrochloric acid. Based on these results, demineralized water was added as needed to the tanks to maintain the desired water CaCO_3_ content during the experiment. Determination of the needed amounts of demineralized water was accomplished by the use of a graph, which was established before the experiment started. Other divalent ions contributing to total water hardness were negligible.

Once a week, nitrite, nitrate, phosphate and ammonium levels were determined by photometric measurement (LASA 100 Photometer, Hach‐Lange GmbH).

Lighting was provided for nine hours daily at true light quality (2× 15‐W T8 Sylvania®, Feilo Sylvania). The duration of the experiment was 16 weeks.

### 2.1 Examination of the fish

Upon arrival, all fish were measured and weighed. Photographs of all fish were taken from both sides of the head, with emphasis of the supra‐ and infraorbital region and the lateral line region with a digital camera (NIKON D300s digital camera, Nikon Corp.). Reference fish (one of each group) were killed and sampled in the same way as the other fish at the end of the experiment. All other fish were acclimated and assigned to the different groups as described above. Photodocumentation was repeated after four, eight and twelve weeks and at the end of the study (16 weeks). During the experiment, all fish were examined daily for the development of lesions indicative of HITH disease. For this examination, fish were gently guided to the front glass of the aquaria with the help of a transparent perforated sheet and carefully inspected. Anticipated macroscopic signs of HITH were depigmentation around and enlargement or confluence of sensory pores, pitting of the skin of the head, and the emergence of white amorphous masses in sensory pores. After 16 weeks, the fish were killed with an overdose of buffered tricaine methanesulphonate (1 g L MS222, Sigma‐Aldrich). A 0.3 ml blood sample was withdrawn immediately from the caudal vein with a heparinized 1‐ml syringe and a 23‐gauge needle and carefully transferred to a 1.5‐ml Eppendorf tube (Eppendorf tubes®, Eppendorf AG). The tubes were immediately cooled to 4°C and proceeded to the Clinical Pathology Platform of the University of Veterinary Medicine of Vienna, where they were centrifuged at 5,000 rpm for 5 min in a bench‐top centrifuge (Centrifuge 5415D). The plasma was separated, and Ca, P and Mg plasma levels were analysed with a blood chemistry analyser (Cobas 6000/c501, Roche), using accredited photometric and colorimetric methods. Since no reference plasma mineral levels for discus are available, values were compared to reference intervals of other fish species. After blood removal, skin and sensory pores of all fish were again carefully inspected and pictures were taken. For bacteriology, swabs from sensory pores and the surrounding skin of all fish were inoculated on Columbia sheep blood (COS) agar plates (Thermo Fisher Scientific, Oxoid Limited) at 26°C. The plates were inspected daily, and growing cultures were subcultured and identified by gram stain, light microscopy and API identification system (BioMerieux). Skin, sensory pores and gills were routinely examined by wet mounts for the presence of parasites. For this, samples of gills, skin (around sensory pores, base of the pectoral fin, base of the dorsal fin) and mucus were gently scraped with a coverslip in an angle of about 45°. Specimens were brought to a slide and identified at 400× magnification with a light microscope (Olympus Microscopes BX 53, Olympus Austria) according to morphological criteria (Hoffman, 1999). Densities of parasites are given per wet mount or field of view at 200× magnification according to Bush, Lafferty, Lotz, and Shostak (1997). All bacteriological and parasitological samples from skin and sensory pores were collected from the right side of the fish, whereas specimens for histopathology were taken from the other side. For histological examinations, biopsy samples from the head (supraorbital region, infraorbital region, nostrils with surrounding tissue) and the trunk (lateral line, 1 cm caudal of the operculum), including cartilage and bones, were taken in triplicates. The tissues were first fixed in neutral buffered formalin. After decalcification (Decal®, SERVA Electrophoresis GmbH) overnight, the tissues were cut and embedded in paraffin wax and 5 µm sections were stained with haematoxylin and eosin (H&E, Eberhard Lehmann GmbH). The prepared slides were labelled with codes to allow a blinded analysis. Histological analysis was focused on sensory pores, lateral line channels and underlying bones. For assessment of histological changes, the following parameters were evaluated by two independent persons: epithelium (infiltration, hyperplasia, hypoplasia, sloughing and necrosis), evidence of exudates, evidence of bone resorption and remodelling and other pathological changes. Changes were scored from 0 (normal appearance) to 3 (severe alteration) and the total score for each group calculated. Following the sampling for histopathology, the abdominal cavity was opened, the macroscopic appearance of the abdominal organs was noted, and the gender of the fish was determined. Organs were removed, and biopsy samples from spleen, liver, kidney and intestinal tract were stored at −80°C for further use if required. Wet mounts from the epithelium of the cranial, the middle and the caudal part of the intestinal tract were prepared, mixed with a drop of tap water and examined immediately under a light microscope at 400× magnification. Additionally, mucosal scrapings from the intestine as indicated above were stored in 2 ml tubes at −20°C to identify prospective diplomonad flagellates by molecular genetic methods.

### 2.2 Statistical analysis

The hypothesis of this study was that a severe imbalance of dietary Ca and P may lead to HITH disease. A plain beef heart diet meets these criteria and was therefore fed to half of the fish in the experiment (*n* = 10). The other half served as a control group and was fed a commercial maintenance diet. Should the hypothesis prove true, all fish of one group should be affected. Thus, the number of used animals was kept low. Besides the mere observation of signs of HITH disease, various quantitative parameters were collected, which allowed a cautious comparing of the groups.

To reveal possible statistical correlations between water parameters, feeding regime and plasma levels of Ca, P and Mg, the following tests were used in IBM® SPSS version 24.0: descriptive statistic to provide the mean and standard deviation for each evaluated parameter, a test of between‐subjects effects to show whether either of the two independent variables (feeding regime and water parameters) or their interaction was statistically significant with reference to Ca, P and Mg. A one‐way ANOVA was conducted to investigate an influence of the different water parameters on the blood levels of Ca, P and Mg. Equal variances were assumed doing the Levene's test for equality of variances. Fisher's LSD post hoc test was applied to test for the significance of the former results.

## 3 RESULTS

**Figure 1:**
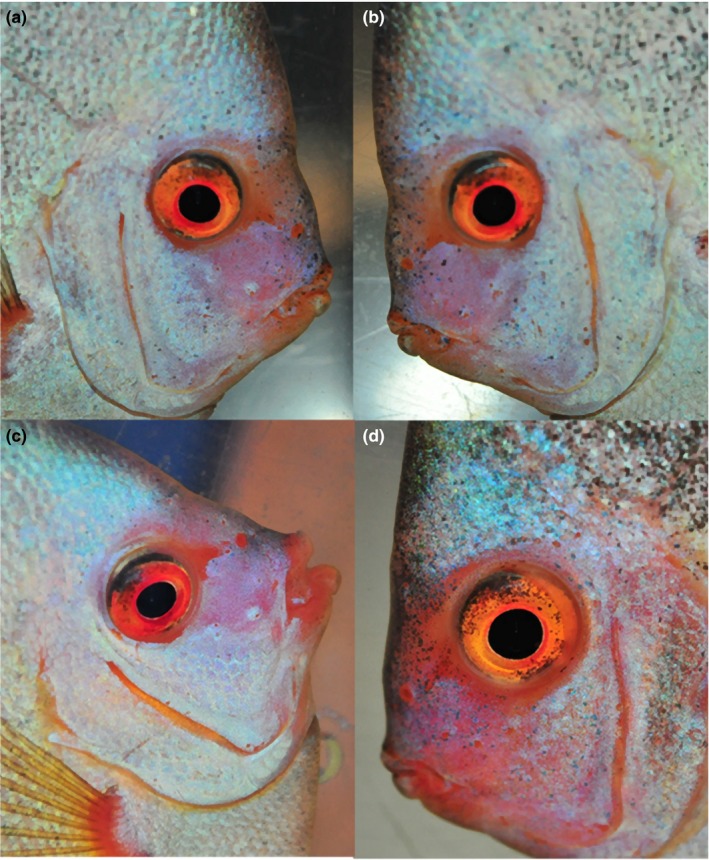
Exemplary discus from beef heart group at the beginning (a and b) and at the end (c and d) of the study. No macroscopic changes (enlargement, confluence, change of pigmentation or exudates) of the sensory pores and no pitting of the skin consistent with HITH developed during the experiment [Colour figure can be viewed at http://www.wileyonlinelibrary.com]

For the duration of the study, no mortalities occurred and all fish fed well. Length gain rates (LGR) were 5% for groups A and C and 5.96% for groups B and D, while weight gain rates (WGR) were 10.16% and 7.76% respectively (Table 1). Macroscopic observations and evaluation of the photographs revealed no lesion development consistent with HITH disease and no obvious changes of sensory pores in any of the fish (Figure 1).

Results of the food analysis are given in Table 2. Both diets were imbalanced regarding their Ca/P contents (Ca/P of 0.03 in the beef heart diet and of 2.73 in the commercial diet).

**Table 2:** Results of diet analysis and dietary requirements regarding Ca, P and Mg (g 1000g^‐1^ wet weight)

	Beef heart (g 1000 g^−1^)	Commercial diet (g 1000 g^−1^)	Dietary requirements (g 1000 g^−1^)
Ca	0.06	20.1	1.8
P	2.06	7.36	1.5
Mg	0.21	1.69	0.5–1.0
Ca/P	0.03	2.73	1.3

Note

As no valid data for discus are available, reference values were taken from Chavez‐Sanchez et al. (2000) for *Cichlasoma urophthalmus* (Günther). Reference values of Mg originate from Kaushik (1999).

No parasites were detected in samples of skin and the sensory pores of any reference or experimental fish. In one fish of group C, one *Dactylogyrus* sp. per wet mount was observed in the gills. A density of 20–50 diplomonad flagellates per field of view at 200× magnification was found in the gut of two fish of group B and of more than 50 per field of view in two fish of group C.

Bacteria isolated from the skin belonged mostly to the orders of Aeromonadales and Pseudomonadales. At the end of the experiment, *Aeromonas* sp. were isolated from all fish except any fish of group B. *Pseudomonas* sp. were found in all groups except for one fish of group B, where *Ochrobactrum anthropi* and *Acinetobacter baumannii* were detected. In fish of group B, also *Proteus vulgaris* was identified (Table S1).

Plasma values for Ca, P and Mg were determined (Table 3). The results of plasma Ca and P of reference fish were lower compared to the results of fish from groups A–D, and this also applies to Ca values of the reference fish compared to other studies (Table 4). Except for group B (commercial diet, low water Ca), Ca values of most experimental fish were lower than reported in other studies, whereas the levels of P of all fish and of Mg of all but two fish (both group D) were higher (Tables 3 and 4).

**Table 3:** Plasma chemistry results for Ca, P and Mg of each food/water quality group and reference fish

Group/food	Water CaCO_3_ mg/L	Plasma Ca mmol/L			Plasma P mmol/L			Plasma Mg mmol/L		
		Mean	Range	*SD*	Mean	Range	*SD*	Mean	Range	*SD*
Reference fish	124.9–196.3	2.09	1.46–2.77	0.49	4.84	4.04–5.29	0.50	n.d.	n.d.	n.d.
A/beef heart	35.7–71.4	2.72	2.34–3.27	0.38	5.64	4.74–6.08	0.60	1.85	1.40–2.20	0.34
B/commercial diet	35.7–71.4	3.47	2.05–5.06	1.08	6.75	4.31–8.15	1.62	1.66	1.18–2.83	0.70
C/beef heart	124.9–196.3	2.33	2.08–2.73	0.26	6.37	5.82–7.53	0.68	1.76	1.62–2.07	0.18
D/commercial diet	124.9–196.3	2.20	1.79–3.02	0.51	6.61	3.61–8.57	2.04	1.15	0.81–1.60	0.29

Abbreviations: *SD*, standard deviation; n.d., not determined.

**Table 4:** Resumed plasma Ca, P and Mg values from all fish of groups A–D and reference fish compared to the results of other studies and former results (healthy discus, HITH diseased discus)

mmol/L	Hrubec, Cardinale, and Smith (2000) (low density)	Mauel et al. (2007)	Snellgrove and Alexander (2011)	Chen, Wooster, Getchell, Bowser, and Timmons (2003)	Tripathi et al. (2005)	This study Groups A–D	This study Reference fish	Healthy fish *n* = 4	HITH diseased *n* = 1
	*Oreochromis hybrid*	*Oreochromis aureus* × *Oreochromis niloticus*	*Metriaclima greshakei*	*Oreochromis niloticus*	*Cyprinus carpio*	*Symphysodon aequifasciatus*	*Symphysodon aequifasciatus*	*Symphysodon aequifasciatus*	*Symphysodon aequifasciatus*
Ca	2.95	7.43	2.6	4.36	2.74	2.68	2.09	2.30	13.64
P	1.49	6.62	1.3–1.6	3.18	1.82	6.34	4.84	2.90	6.14
Mg	1.03	2.01	n.d.	1.20	1.23	1.61	n.d.	n.d.	*n*.d.

Note

All values are given as means.

Abbreviation: n.d., not determined.

All histological slides were scored 0 by both investigators, which means that none of the anticipated changes could be observed. The multilayered squamous epithelium contained mucous cells, eosinophilic granular cells, lymphocytes, macrophages and superficial neuromast cells. The superficial neuromast cells were enclosed by supporting cells. Sensory pores were identified in the epidermis. The sensory pores were lined by a single‐layered squamous epithelium. Sensory canals, containing the deep neuromast cells, were found in the stratum spongiosum of the dermis. They were encompassed by connective tissue or bone, respectively, and lined by single to multilayered epithelium which contained eosinophilic granular cells. The lateral line canal of the trunk was composed of short connected sections and lined by a multilayered squamous epithelium, also containing eosinophilic granular cells. Compared to the head region, the epidermis and dermis were quite thinner on the trunk. The microscopic examination of the bones revealed acellular bone without osteocytes, but a structural layering arrangement could be clearly seen. Unaffected chondrocytes surrounded by a matrix of collagenous fibres could be identified in cartilage tissue. Scales showed a homogenous stratified structure with sawtooth‐like ridges.

### 3.1 Statistical results

The results of the descriptive statistics demonstrated the highest mean plasma value of Ca and P in group B (commercial discus food, low water Ca), whereas the highest mean value of the plasma levels of Mg was detected in group A (fed beef heart, low water Ca). The test of between‐subjects effects revealed no statistically significant interaction between feeding regime and blood parameters (Table S2). There was a significant effect of water CaCO_3_ on plasma Ca levels (*p* = 0.010) in any group. Accordingly, the one‐way ANOVA showed a statistically significant difference of plasma Ca levels between the different levels of CaCO_3 _in the water (*p* = 0.012). Finally, Fisher's LSD post hoc test illustrated a significant difference of mean plasma Ca levels between groups B and C (*p* = 0.012) and groups B and D (*p* = 0.006) and of Mg between groups A and D (*p* = 0.020) and groups C and D (*p* = 0.039) (Table S3). Still, these results are biased by the high *SD* (>1) in group B for Ca and the small number of specimens use. No significance was revealed for P. In summary, the feeding regime did not cause statistically significant differences of Ca, P and Mg plasma levels between the groups. Water CaCO_3_ did cause a significant difference in plasma Ca.

## 4 DISCUSSION

The study was conducted to investigate whether an imbalanced beef heart diet could cause HITH disease in discus fish. In this context, several additional parameters were evaluated to improve assessment of the condition. During the experiment, no signs of HITH disease were evident in any fish despite the unfavourable Ca/P ratio of both diets. The growth rate of the fish in all groups was rather low when compared with other studies (Tibile et al., 2016), despite good food intake. While the growth rate did not differ substantially between the groups, weight gain was higher in the beef heart groups. In a study of Wen, Chen, Qu, and Gao (2018), discus on a plain beef heart diet showed significant higher WGR (90.33%) and LGR (60.84%) than in our study. These differences can be explained by different feed quantities and the age of the fish, assumingly plus other environmental factors. Moreover, the small number of fishes per group did not allow a statistical sound statement regarding growth and weight gains.

In context with a publication by Peyghan, Boloki, and Ghorbanpour (2010) who described HITH disease associated with the isolation of *Aeromonas hydrophila*, bacteria were cultured from the sensory pores and the surrounding skin of each fish in our experiment. All isolated bacteria represented only a few orders (Table S1) and can be found in waters and biofilms as opportunistic microbes (Drzewiecka, 2016; Walczak, Puk, & Guz, 2017). Despite their ability to cause diseases and mortality of fish under favourable conditions (Austin & Austin, 2016), this did not apply to any fish in this experiment. However, it is notable that the bacteria isolated from fish from group B differed considerably in their species composition compared to the other groups. It must be assumed that fish of all groups showed a similar composition of bacteria upon arrival. This assumption is supported by isolates from the reference fish (Table S1). Thus, either a shift of the bacterial population or the introduction of bacteria during the experiment might have happened, which we consider of no significant importance for the experiment.

**Figure 2:**
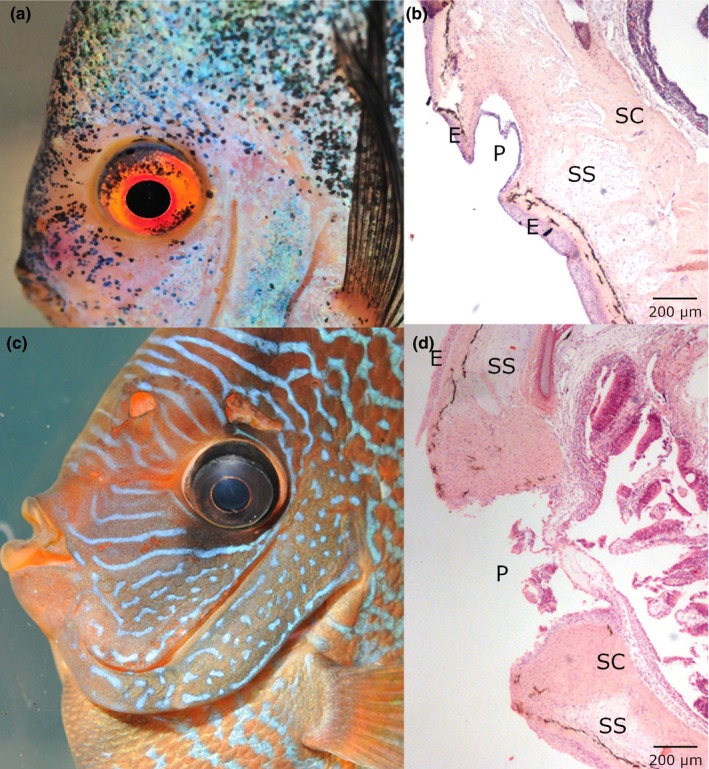
(a) Discus from group C, no lesions of HITH disease are evident. (b) Histology (HE stain) from the same fish. Skin covering the olfactory organ in the rostral region of the eye. Multilayered epithelium (E) is covering the skin, which changes to a single layer, lining the sensory pore (P) (lift of epithelium is an artefact). Stratum spongiosum (SS) and stratum compactum (SC) of the dermis can be identified. (c) HITH disease‐affected discus for comparison (not from this study). Lesions of the sensory pores of varying size can be seen. (d) Histology (HE stain) from the same fish. Skin, covering the olfactory organ in the rostral region of the eye. The epithelial lining of the sensory pore has been lost. In the centre of the pore, an accumulation of fibroblasts, eosinophilic granular cells and leucocytes can be found. A small line of basal cells and connective tissue divides the olfactory organ from the skin lesion [Colour figure can be viewed at http://www.wileyonlinelibrary.com]

Parasitic examinations were included in the study because parasitic infestation may serve as an indicator for a compromised health status in general (Williams et al., 2013) but also may cause disease itself, thus being an important unspecific factor for the anticipated development of HITH disease. On the other hand, protozoa belonging to the genus *Spironucleus* have been suggested as the causative agent of HITH disease (Bassleer, 1983; Herkner, 1969, 1970; Paull & Matthews, 2001). Except for one fish of group C (*Dactylogyrus* sp. in the gills), no external parasites were detected in fish from this study. Diplomonad flagellates were not detected in the gastrointestinal tract of the reference fish, but in two fish of group B and two of group C. This was not an unexpected finding, as flagellates have been suggested as putative intestinal commensals in cichlids such as angelfish, discus, oscars and African cichlids (Woo & Poynton, 1995). On the other hand, the occurrence of vast numbers of *Spironucleus* sp. in the gut may lead to severe enteritis and has the potential of invading host tissue and inducing systemic infection (Williams et al., 2013). Such a systemic infection with *Spironucleus vortens* has been linked to HITH disease with parasites recovered from internal organs and skin lesions of diseased fish, and it has been suggested that the infection originated from the intestinal tract where trophozoites of the flagellate were demonstrated (Paull & Matthews, 2001). In our study, the presence of diplomonad flagellates in the intestinal tract of four fish did not result in intestinal signs or lesions or the development of HITH disease. Given the fact that no signs of HITH disease could be observed macroscopically and by photography, special care was taken in examining the histological slides. No changes in epithelial tissue of the skin and the sensory canals, the neuromast cells or cartilage and bone structures were identified, and no evidence of HITH disease as described by Morrison, O'Neil, and Wright (2007) was observed. The macroscopic and histological comparison of healthy and HITH disease‐affected discus fish is shown in Figure 2. To demonstrate a possible connection between selected plasma values and the development of HITH disease, the analysis of Ca, P and Mg values was included in the study. Haemolysis, which may result in increased levels of Ca, P and Mg (Mirghaed, Ghelichpour, Hoseini, & Amini, 2017), was prevented by appropriate handling of the samples. MS 222 anaesthesia should not impact Ca, P and Mg levels (Velíšek, Stejskal, Kouřil, & Svobodová, 2009). Among environmental factors, the water Ca contents and the availability of dietary P are mostly responsible for meeting the requirements of the two minerals in fish (Nose & Arai, 1979). Discus are endemic to the Amazon River Basin and inhabit black water streams as well as white and clear water regions. Water chemistry, including Ca contents of these waters, differs considerably, and it has been shown that the Ca content of tail vertebrae from discus differed accordingly to their habitat (Geisler & Schneider, 1976). Still, our study relies on plasma values, since they reflect actual metabolic activity as observed in secondary hyperparathyroidism. Moreover, blood is easily accessible, even from live fish. The different feeding regimes did not result in statistically significant different plasma values of the tested minerals. However, Ca concentration in the water had an influence on plasma Ca levels, albeit the fish in groups with low water Ca had higher plasma Ca levels. Nevertheless, this result must be interpreted cautiously, since the number of specimens was low and a single statistical outlier (one fish of group B) had a disproportionate influence on these results. The same is true for the interpretation of the statistical comparison of Ca and Mg levels of the different groups; to soundly confirm the differences, a higher number of samples would have been required. Nevertheless, comparing the results of all measured plasma levels, they show a quite homogenous picture with similar Ca and high P levels when compared to the references as given in Table 4. The high P results from our study are only in accordance with the results of Mauel, Miller, & Merrill, 2007. These authors contribute the higher values of some of the parameters in their study to high stocking density and differences between species/strains. Our discus were stocked at low densities, and the high plasma P levels most likely result from the excessive amounts of P in the diets, which contrasts with the much lower natural intake of the mineral with insects as the main source of protein in the wild (Bleher, 2010). We also compared our values with former results from four healthy discus, which were fed a mixed diet and found comparable results for Ca and Mg, whereas the value of P was much lower in this group (Table 4), which gives an additional indication that the high P contents of the diets resulted in high plasma values of the mineral. Otherwise, the high plasma P values were comparable to the archived results from a HITH diseased discus, whereas this fish also had a high plasma Ca content. In conclusion, our study indicates that an unfavourable Ca/P in the diet of discus may result in altered P plasma levels and justifies further research with larger numbers of animals to verify these results. On the other hand, an unfavourable Ca/P in the diet did not provoke HITH disease or evidence of bone resorption or remodelling during the observation period under the selected environmental conditions. Thus, HITH disease was not observed in discus fed a plain beef heart diet.

## CONFLICT OF INTEREST

The authors declare no conflict of interest.
