# Supplementary material for: Hole‐in‐the‐head disease in discus fish, Symphysodon (Heckel, 1840): Is it a consequence of a dietary Ca/P imbalance?
Source: J Fish Dis. 2019 May 26;42(8):1133–42. doi: 10.1111/jfd.13023 (PMC6852440; doi:10.1111/jfd.13023)
Supplement: Supplementary file 1 [file JFD-42-1133-s001.docx]

**Supporting material**

**Table 1**

Cultivated bacteria from sensory pores and surrounding skin from reference (ref, n=1 per group) and experimental (exp, n=5 per group) fish.

| Fish | Order of bacteria | | | |
| --- | --- | --- | --- | --- |
| Group/type | Aeromonadales | Pseudomonadales | Enterobacteriales | Others |
| A/ref | *------* | ------ | ------ | ------ |
| A/exp | *A. hydrophila* | *P.putida* | ------ | ------ |
| B/ref | *------* | *P. aeruginosa* | ------ | ------ |
| B/exp | *------* | *P. aeruginosa*  *P. luteola*  *P. alcaligenes*  *P. putida* | *Proteus vulgaris* | *Ochrobactum anthropi*  *Acinetobacter baumannii* |
| C/ref | *A. sobria* | *------* | ------ | ------ |
| C/exp | *A.sobria* | *P. luteola*  *P. aeruginosa* | ------ | ------ |
| D/ref | *A.sobria* | *------* | ------ | ------ |
| D/exp | *A.sobria*  *A. hydrophila* | *P. putida* | ------ | ------ |
