# Supplementary material for: Hole‐in‐the‐head disease in discus fish, Symphysodon (Heckel, 1840): Is it a consequence of a dietary Ca/P imbalance?
Source: J Fish Dis. 2019 May 26;42(8):1133–42. doi: 10.1111/jfd.13023 (PMC6852440; doi:10.1111/jfd.13023)
Supplement: Supplementary file 2 [file JFD-42-1133-s002.docx]

**Table 2**

**Results of Test of Between Subjects Effects; significance p<0.05.**

| **Subjects** | **Blood Parameters** | **Significance** |
| --- | --- | --- |
| Food | Calcium | P=0.296 |
|  | Phosphorus | P=0.293 |
|  | Magnesium | P=0.053 |
| Water parameters | Calcium | P=0.010 |
|  | Phosphorus | P=0.644 |
|  | Magnesium | P=0.136 |
