# Supplementary material for: Hole‐in‐the‐head disease in discus fish, Symphysodon (Heckel, 1840): Is it a consequence of a dietary Ca/P imbalance?
Source: J Fish Dis. 2019 May 26;42(8):1133–42. doi: 10.1111/jfd.13023 (PMC6852440; doi:10.1111/jfd.13023)
Supplement: Supplementary file 3 [file JFD-42-1133-s003.docx]

**Table 3**

**post-hoc Fisher´s LSD Test: multiple comparisons of mean values of Ca, Mg and P between groups, significance p<0.05.**

| **Blood parameters** | **Group** | **Group** | **Significance** |
| --- | --- | --- | --- |
| Ca | Group A | Group B | 0.083 |
|  |  | Group C | 0.347 |
|  |  | Group D | 0.216 |
|  | Group B | Group A | 0.083 |
|  |  | Group C | 0.012 |
|  |  | Group D | 0.006 |
|  | Group C | Group A | 0.347 |
|  |  | Group B | 0.012 |
|  |  | Group D | 0.752 |
|  | Group D | Group A | 0.216 |
|  |  | Group B | 0.006 |
|  |  | Group C | 0.752 |
| Mg | Group A | Group B | 0.488 |
|  |  | Group C | 0.738 |
|  |  | Group D | 0.020 |
|  | Group B | Group A | 0.488 |
|  |  | Group C | 0.716 |
|  |  | Group D | 0.078 |
|  | Group C | Group A | 0.738 |
|  |  | Group B | 0.716 |
|  |  | Group D | 0.039 |
|  | Group D | Group A | 0.020 |
|  |  | Group B | 0.078 |
|  |  | Group C | 0.039 |
| P | Group A | Group B | 0.223 |
|  |  | Group C | 0.417 |
|  |  | Group D | 0.287 |
|  | Group B | Group A | 0.223 |
|  |  | Group C | 0.669 |
|  |  | Group D | 0.869 |
|  | Group C | Group A | 0.417 |
|  |  | Group B | 0.669 |
|  |  | Group D | 0.792 |
|  | Group D | Group A | 0.287 |
|  |  | Group B | 0.869 |
|  |  | Group C | 0.792 |
